# Diagnostic value of mid-regional pro-Adrenomedullin as a biomarker of invasive bacterial infection in children: a systematic review

**DOI:** 10.1186/s12887-022-03255-9

**Published:** 2022-04-04

**Authors:** Michael Paul Corr, Derek Fairley, James P. McKenna, Michael D. Shields, Thomas Waterfield

**Affiliations:** 1 Centre for Public Health, Queen’s University Belfast, Belfast, UK; 2 Department of Microbiology, Belfast Health and Social Care Trust, Belfast, UK; 3 Centre for Experimental Medicine, Wellcome Wolfson Institute of Experimental Medicine, Queen’s University Belfast, Belfast, UK

**Keywords:** Adrenomedullin, MR-proADM infection, Paediatrics, Bacterial infection, Biomarkers

## Abstract

**Background:**

Invasive bacterial infections (IBI) in children present a difficult clinical challenge. They are often life-threatening, however in the early stages they can be hard to differentiate from benign viral infections. This leaves clinicians with the risk of missing a serious IBI diagnosis or inappropriately using antimicrobials in a child with a viral infection- contributing to the ongoing development of increased antimicrobial resistance. Hence, biomarkers which could aid in early detection of IBI and differentiation from viral infections are desirable. Mid-Regional pro-Adrenomedullin (MR-proADM) is a biomarker which has been associated with IBI. The aim of this systematic review was to determine its diagnostic accuracy in identifying children with IBI.

**Methods:**

A strategy was devised to search online databases MEDLINE, Embase, Web of Science and Scopus for human clinical trials reporting the accuracy of MR-proADM in children. Against predesigned inclusion and exclusion criteria full texts were selected for inclusion and data extraction. True positives, false positives, true negatives and false negatives were extracted from each included study to fill 2 × 2 tables. Using the Quality Assessment of Diagnostic Accuracy Studies (QUADAS-2) tool methodological quality of each study was assessed.

**Results:**

A total of 501 articles were initially identified. After the removal of duplicates and abstract screening 11 texts were fully reviewed and four texts (totaling 1404 patients) were included in the systematic analysis. Only one study was of a high quality and that study accounted for the vast majority of patients. A single study reported the diagnostic accuracy of MR-proADM for invasive bacterial infection reporting an Area under the Curve of 0.69. The paucity of available studies made meta-analysis and studies of heterogeneity impossible.

**Conclusion:**

There is a paucity of research regarding the diagnostic accuracy of MR-proADM in the diagnosis of invasive bacterial infections in children. Initial results would suggest that MR-proADM testing alone is poor at identifying IBI in young children. It remains unclear if MR-proADM performs differently in older children or in children with signs and symptoms of IBI.

**Trial registration:**

PROSPERO CRD42018096295.

**Supplementary Information:**

The online version contains supplementary material available at 10.1186/s12887-022-03255-9.

## Background

Invasive bacterial infections (IBI) are defined as the identification of pathogenic bacteria from a sterile fluid or body tissue by either culture or molecular diagnostic techniques such polymerase chain reaction (PCR). Worldwide IBIs are the leading cause of neonatal and paediatric morbidity and mortality [1]. As many as one in three neonatal deaths globally are attributed to bacterial sepsis and meningitis [2, 3]. The early detection and treatment of IBI is crucial to prevent poor clinical outcomes for patients [4]. However, differentiating between IBIs and self-limiting viral infections remains diagnostically challenging. When there is diagnostic uncertainty clinicians typically perform additional investigations including blood tests. The results of these tests are used to guide treatment decisions including the use of antimicrobial medications [4].

Biomarkers for severe infections or IBI are often measured from plasma samples taken at i) presentation to hospital to initiate treatment ii) as an inpatient to guide treatment and iii) to prognosticate on expected outcomes including the need for Intensive Care Unit (ICU) admission. In current clinical practice C-Reactive Protein (CRP) and Procalcitonin (PCT) are the most widely used biomarkers to used detect IBI and monitor response to treatment [5]. Unfortunately, both CRP and PCT lack the necessary accuracy to reliably detect IBI in the early stages [5]. This diagnostic uncertainty requires many clinicians to prescribe antimicrobial medications for febrile children “just in case”. This cautious, but necessary approach, has resulted in many children with self-limiting viral illnesses receiving broad-spectrum antimicrobial therapy. The widespread use of antimicrobial drugs leads to increased anti-microbial resistance which is a growing public health problem- in Europe an estimated 30% of paediatric bacterial infections are due to multidrug resistant bacteria [6]. Novel biomarkers that are better at differentiating children with IBI could significantly improve clinical care for paediatric patients presenting with a fever by reducing the need for admission, reducing the use antimicrobial drugs and improving antimicrobial stewardship.

The index test for this systematic review is Mid-Regional pro-Adrenomedullin (MR-proADM). MR-proADM is a peptide that is related to adrenomedullin (ADM). ADM was first isolated and detected in 1993 and has been found to be involved in a wide range of human physiological processes [7]. ADM acts as a circulating hormone with paracrine activity that effects the vasculature. The normal range for ADM in healthy individuals is 2 to 4 pmol/l. ADM levels are elevated in patients with bacterial infections, with initial data suggesting that ADM levels may rise before other commercially available biomarkers [8]. ADM levels have also been shown to rise in response to other noxious stimuli such as hypoxia or the release of cytokines such as Interleukin-1, Interferon-ϒ and Tumour Necrosis Factor (TNF) [9–12]. ADM levels have been shown to correlate with disease severity and have been shown not to rise significantly in isolated viral infections [13–16].

Although ADM has near ideal characteristics as a biomarker of IBI its use in clinical practice has been limited. This is due to the difficulty in reliably measuring ADM levels in-vivo [16]. ADM is an unstable protein with rapid binding to receptors, fast metabolism and a short half-life [11].

MR-proADM, a 48 amino acid fragment that is produced in conjunction with ADM at a ratio 1:1 is a significantly more stable molecule making it easier to measure in-vivo [13]. There is no known biological role of MR-proADM and it can be used as a surrogate measure of ADM levels.

Research assessing MR-proADM as a predictive diagnostic biomarker has been increasing in recent years with researchers investigating its role in paediatrics. A recent systematic narrative review suggested that MR-proADM may have a diagnostic role in the diagnosis of serious bacterial infections (non-IBI) in children as well as in non-infective inflammatory conditions such as juvenile arthritis [17]. However, a systematic review is required to determine if sufficient research exists to describe the role of MR-proADM in the diagnosis of IBI in children. If found to have a role in IBI diagnosis it could be investigated for further uses such as; monitoring of response to antimicrobial treatment and to prognosticate on the need for intensive care. Reducing the use of parenteral antimicrobial agents has the potential to reduce antimicrobial resistance. Furthermore, serial measurements of MR-proADM could be used to monitor the response to treatment or predict those requiring ICU admission.

The aim of this review was to establish the diagnostic accuracy of MR-proADM in the detection of IBI in children under the age of 18. The secondary aim was to determine the diagnostic accuracy of MR-proADM in various age-related subgroups such as newborns, neonates, children and adolescents and whether any differences in accuracy or optimal cutoff values existed between these groups.

## Methods

### Protocol and registration

Prior to conducting this systematic review a protocol was produced in adherence to the standards of the Preferred Reporting Items for Systematic Reviews and Meta-Analyses (PRISMA) and registered prospectively on the 30/05/2018 with the International Prospective Register of Systematic Reviews (PROSPERO) - registration number CRD42018096295. The protocol underwent external peer review and was published in 2020 [18].

### Eligibility criteria

All case-control studies, cohort studies and randomised control trials reported in any language that assess the performance of MR-proADM in assessing children (< 18 years of age) for potential IBI were considered. The index test was MR-proADM performed on any bodily fluid (including but not limited to blood/urine/cerebrospinal fluid) using commercially and non-commercially available tests. The reference standard was positive blood or cerebrospinal fluid culture or PCR for pathogenic bacterial infection taken at the same time as the index text.

### Information sources

An electronic search strategy was developed in collaboration with the Queen’s University Belfast Medical Librarian (RF). MEDLINE, Embase, Web of Science, Scopus and the Cochrane Library inclusive of Cochrane Controlled Trials Register were searched from inception to 29th of November 2021. The Medline search strategy is attached in the supplementary material. There were no language restrictions. A targeted grey literature search was also be conducted by review of clinical trials databases, conference abstracts, internet searches and review articles. Mendeley electronic reference manager was used for article retrieval.

### Study selection and data extraction

Two reviewers (TW, MC) independently screened all abstracts and titles against inclusion criteria and assessed full text publications for eligibility. The same two reviewers independently judged study quality using the Quality Assessment of Diagnostic Accuracy Studies (QUADAS-2) tool [19]. Disagreements were resolved by consensus or arbitration by a third party (JMK). Using a pre-piloted data extraction tool (supplementary material), two reviewers (MC, JMK) independently extracted the following information:

- Study characteristics: author, year of publication, country, design, sample size, clinical setting, number studied, number of drop-outs with reason, and funding source.
- Population characteristics: inclusion/exclusion criteria; patient demographics
- MR-proADM Testing: timing of sampling; method of sampling
- Gold standard: Real-time PCR (e.g.TaqMan® PCR) or sterile site bacterial culture (i.e blood/cerebrospinal fluid)
- Outcomes: True positives, false positives, true negatives, and false negatives were extracted to construct a diagnostic contingency (2-by-2) table.

Where data was unavailable or incomplete the authors were contacted and asked for additional data and/or clarification of results.

### Analysis

Statistical analysis and data synthesis were performed by TW and MC. MR-proADM test result data were compared to the reference test. The true positive, true negative, false positive and false negative rate were recorded and used to create a 2 × 2 tables where possible. From these tables inferred statistics were calculated including sensitivity and specificity with 95% confidence intervals. Meta-analysis to provide pooled sensitivity and specificity data were not performed due to the small number of studies available. Similarly studies of heterogeneity and sub-group analysis were not possible. All analysis was performed using Review Manager (RevMan) Version 5.3. Copenhagen: The Nordic Cochrane Centre, The Cochrane Collaboration, 2014.

## Results

### Study selection

**Fig. 1:**
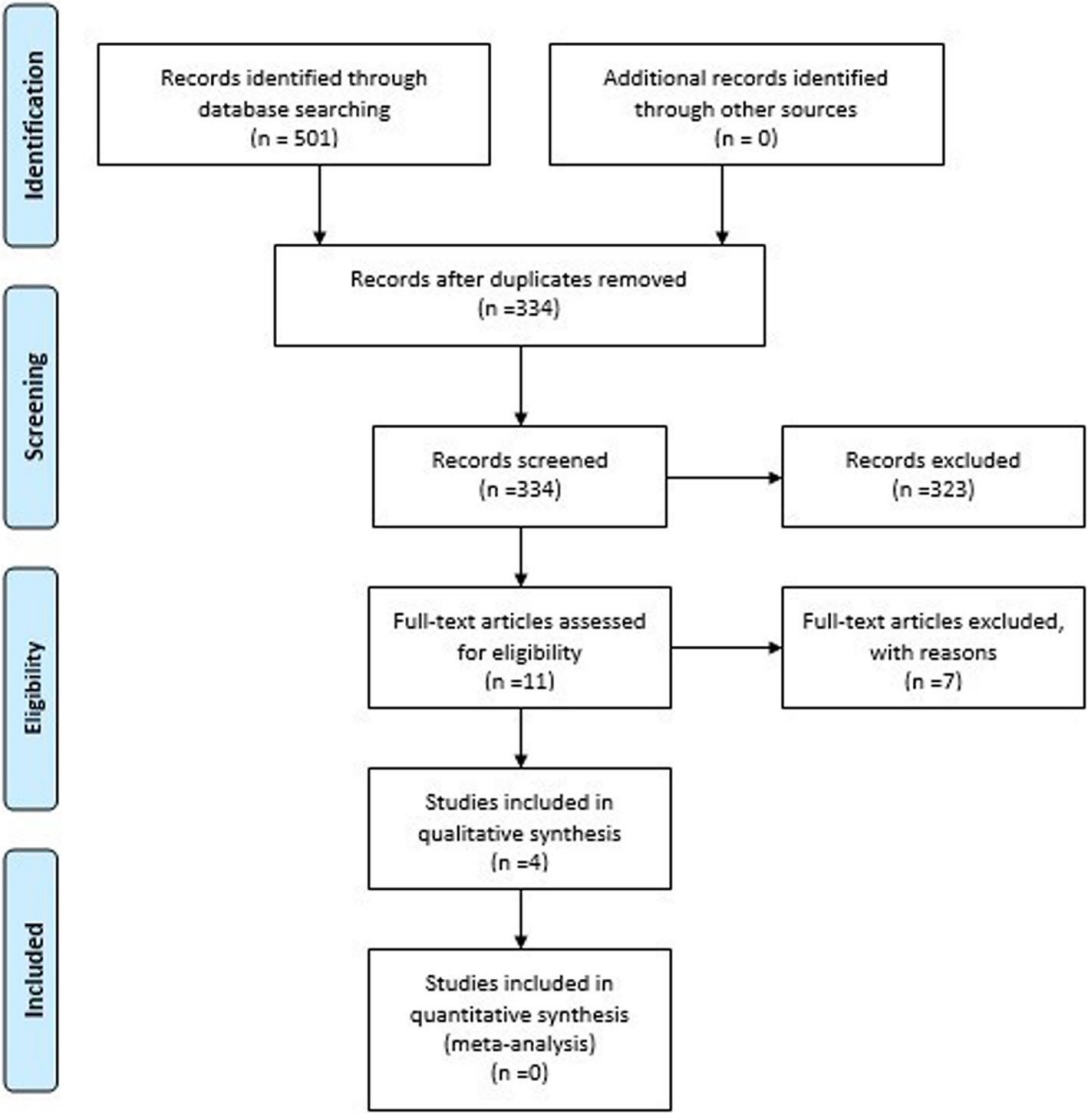
Flow diagram summarizing study selection

A total of 501 records were identified: 501 records from the electronic databases and 0 additional studies from the grey literature. After removal of duplicates, 334 studies were screened, and 323 studies excluded based on the title/abstract. All of the 323 studies screened and excluded were not relevant to the systematic review. There were 11 full text articles that underwent full review, and four studies were eligible for inclusion in the final systematic review [20–31]. Of the seven excluded studies six were excluded because the index test was not MR-proADM and one study was excluded because it only reported the differences in cord blood concentrations of MR-proADM in newborns with and without risk factors for infection [26–31]. Two of the six eligible studies reported on adult and paediatric data [24, 25]. The authors were contacted for any paediatric specific data, but they did not respond [25]. The results of the search with exclusions are summarised in the flow diagram below (Fig. 1).

### Study characteristics and risk of bias

**Fig. 2:**
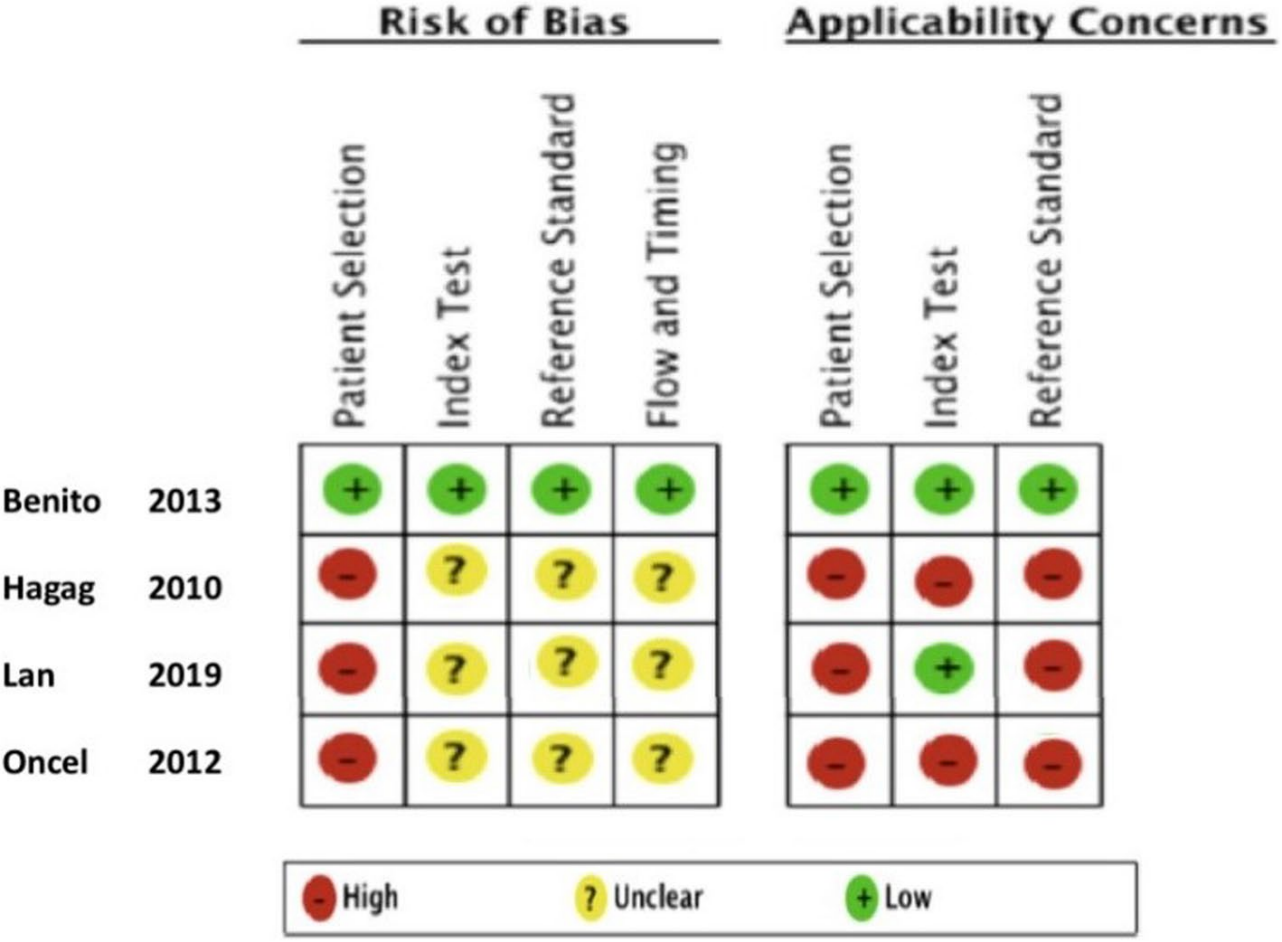
Summary study quality and risk of bias

Four studies including 1404 patients aged between day one of life and 12 years were included in the final systematic review [20–23]. One study was a prospective cohort [23] study and the other three studies were all case-control studies [20–22]. The single prospective cohort study was the largest (*n* = 1077) and the only one to assess the diagnostic test accuracy of MR-proADM for predicting invasive bacterial infection [23]. The three smaller case-control studies (combined *n* = 331) used differing definitions of sepsis as their reference standards [20–22]. These characteristics are summarised in Table 1. The methodological quality of the studies was judged using the QUADAS2 tool. Only the study by Benito et al. was deemed to be applicable and at a low risk of bias [23]. The three case-control studies were all deemed to be a high risk of bias and poorly applicable to the review question (Fig. 2) [20–22].

**Table 1:** Study characteristics for included studies

AUTHOR	YEAR PUBLISHED	NUMBER OF PATIENTS	COUNTRY	DESIGN	CLINICAL SETTING	FUNDING	DROPOUTS	INCLUSION CRITERIA	EXCLUSION CRITERIA	AGE RANGE	TIMING OF SAMPLING	REFERENCE STANDARDS
BENITO	2013	1077	Spain	Prospective cohort study	Paediatric ED	Industry	42	Fever without source	Focal infection Prior antibiotic therapy Immunodeficiency	1 to 36 months of age	Prior to antibiotics	Culture/PCR from sterile site
HAGAG	2010	60	Egypt	Case-control	Neonatal Intensive Care	None declared	0	20 “Mild Sepsis” 20 “Severe Sepsis” 20 “Controls”	None reported	New-borns	Prior to antibiotics	Severity of sepsis
LAN	2019	139	China	Case-control	Intensive Care	State funded	0	94 “Sepsis” 25 “SIRS”^a^ 20 “Controls”	Severe chronic disease Diabetes Specific medications Burns Myocardial infarction Heart failure Rheumatic disease Death within 24 h of admission Incomplete data sets	6 to 12 years of age	Within one hour of admission to intensive care	Severity of sepsis
ONCEL	2012	128	Spain	Case-control	Neonatal Intensive Care	No declared	4	31“Proven Sepsis” 45“Clinical Sepsis” 52 “Controls”	Maternal heart failure or preeclampsia Intracranial bleed	New-borns	Within 6 h of diagnosis of sepsis	Proven sepsis Clinical sepsis

^a^*SIRS* Systematic Inflammatory Response Syndrome

### Results of individual studies

Only the study by Benito et al. (*n* = 1077) provided data directly assessing the diagnostic test accuracy of MR-proADM for the identification of invasive bacterial infection in children [23]. They prospectively assessed 1077 consecutive children under three years of age with fever without source. They reported data on 1035 children with 16 confirmed invasive bacterial infections. In the study by Benito et al. MR-proADM had a reported area under the curve (AUC) of 0.69 (95%CI 0.54–0.85). Benito et al. reported that the diagnostic cut-off for MR-proADM was 0.70 nmol/l [23].

The studies by Oncel et al. (*n* = 128) and Hagag et al. (*n* = 60) were both performed in newborn populations [20, 21]. These case-control studies both demonstrated that MR-proADM levels were higher in the sepsis groups when compared to controls. The study by Hagag et al. reported a correlation between MR-proADM levels and death (*r* = 0.67 *p* < 0.05) [21].

The study by Lan et al. reported that MR-proADM levels were significantly higher in children with paediatric sepsis in the intensive care unit compared to healthy controls (*p* < 0.05). They also reported that the MR-proADM level was positively correlated with severity of sepsis (*r* = 0.62 *p* < 0.05) [22].

The paucity of available studies made meta-analysis and studies of heterogeneity impossible.

## Discussion

### Summary of evidence

The systematic review was primarily designed to determine the accuracy of MR-proADM at identifying invasive bacterial infection in children less than 18 years of age. The review included four studies of 1404 patients [19–22]. Only one study was of a high quality and that study accounted for the vast majority of patients (*n* = 1035) [23]. The remaining studies were all of low quality due to their case-control design and lack of adherence to STARD criteria. From the available literature there is evidence that MR-proADM levels are elevated in cases of sepsis when compared to healthy controls and that levels are correlated with severity. The only study reporting the diagnostic accuracy of MR-proADM for invasive bacterial infection reported an AUC 0.69 [23]. This would suggest that MR-proADM is poor at identifying invasive bacterial infection in children. This would mirror the literature related to MR-proADM in adult populations where there have been significantly more studies. Reviews of MR-proADM in adult populations have not been able to demonstrate utility as a diagnostic tool for IBI but rather as a predictor of clinical sepsis and referral to intensive care units [31].

### Potential of MR-proADM as a biomarker for IBI

Many potential novel biomarkers have been studied in children at risk of IBI, however most do not demonstrate better diagnostic accuracy than the traditionally used biomarkers of CRP and PCT [31]. Some novel markers show potential use in certain settings but a more limited role in others; for example, CD64 (a protein expressed on neutrophils) has been found to be a good diagnostic marker in neonatal sepsis but performed poorly in studies of febrile children [32, 33]. This highlights the importance of conducting validation studies in a wide range of settings. Hence, the secondary aim of this review was to determine, via subgroup analyses, whether the diagnostic accuracy of MR-proADM differs between newborns, neonates, infants, children and adolescents and if different optimal cutoff values exist between different age groups. Similar to the primary objective unfortunately there were insufficient high-quality studies to sufficiently answer this important question.

### Prognostic tool uses/ clinical scoring systems

Increasingly there is an argument that individual biomarkers may have limited diagnostic utility in detecting IBI. Some novel biomarkers have been included in clinical scoring systems in combination with physical findings to improve diagnostic accuracy [34]. Whilst others have been found to be more useful in prognostication in the setting of IBI [35]. In adult populations studies have suggested combining MR-proADM with procalcitonin measurements may have high diagnostic yield and help guide prognosis in the setting of septic shock [36, 37]. In October 2019 the UK’s National Institute of Clinical Excellence developed a briefing on MR-proADM where they highlighted MR-proADM as a potentially effective test for predicting outcomes in adults with sepsis when used with clinical scorings systems [38]. There may be a similar role for MR-proADM in the detection of IBI through scoring systems or as a prognostic tool potentially identifying children in need on intensive care level treatment. Studies identified during this systematic review suggest this as a potential role for MR-proADM in the clinical pathway for children with IBI. Hagag et al., [21] were able to demonstrate correlation between MR-proADM and death related to sepsis in newborns, whilst Lan et al. [22] demonstrated MR-proADM levels as predictive to severity of sepsis. This would be consistent with findings in adults where MR-proADM has been found to related to endothelial damage representing end-organ damage in severe disease states such as septic shock or severe COVID-19 infection [39]. Further studies will be required to fully elucidate if MR-proADM could be used in children as a predictor of requiring intensive care input or as part of a severity scoring system in children with suspected sepsis.

### Limitations

The numbers of studies reporting on the test accuracy of MR-proADM for the diagnosis of invasive bacterial infections in children are small and there is only one high quality paediatric study with a small number of cases of IBI (*n* = 16). The available studies suggest that MR-proADM may have a role in identifying and stratifying sepsis in children, but further studies are required to understand the clinical utility of the test.

## Conclusions

There is a paucity of research regarding the diagnostic accuracy of MR-proADM in the diagnosis of invasive bacterial infections in children. Initial findings indicate that MR-proADM is not a good biomarker for the diagnosis of IBI although further research is required. Specifically, research is required to investigate if MR-proADM has a role in predicting the need for ICU admission in children admitted to hospital with severe infection.

## Supplementary Information

**Additional file 1.**

## Data Availability

All of the individual participant data collected during this study will be available (including data dictionaries) on the Queen’s University Belfast data repository. https://pure.qub.ac.uk/en/datasets/

## Abbreviations

ADM: Adrenomedullin
AUC: Area under the curve
CRP: C-reactive protein
IBI: Invasive bacterial infection
MR-proADM: Mid-Regional pro-Adrenomedullin
PCR: Polymerase chain reaction
PCT: Procalcitonin
