# Supplementary material for: Diagnostic value of mid-regional pro-Adrenomedullin as a biomarker of invasive bacterial infection in children: a systematic review
Source: BMC Pediatr. 2022 Apr 4;22:176. doi: 10.1186/s12887-022-03255-9 (PMC8977188; doi:10.1186/s12887-022-03255-9)
Supplement: Supplementary file 1 — Additional file 1. [file 12887_2022_3255_MOESM1_ESM.doc]

Medline Search Strategy:

Database(s): **Ovid MEDLINE(R) ALL**1946 to November 15. 2021 
Search Strategy:

| **#** | **Searches** | **Results** |
| --- | --- | --- |
| 1 | Adrenomedullin/ | 2760 |
| 2 | adrenomedullin*.mp. [mp=title, abstract, original title, name of substance word, subject heading word, floating sub-heading word, keyword heading word, protocol supplementary concept word, rare disease supplementary concept word, unique identifier, synonyms] | 3425 |
| 3 | MR-proADM.mp. | 238 |
| 4 | "mid-regional proadrenomedullin*".mp. [mp=title, abstract, original title, name of substance word, subject heading word, floating sub-heading word, keyword heading word, protocol supplementary concept word, rare disease supplementary concept word, unique identifier, synonyms] | 19 |
| 5 | exp Bacterial Infections/ | 837780 |
| 6 | "invasive bacteria* infection*".mp. [mp=title, abstract, original title, name of substance word, subject heading word, floating sub-heading word, keyword heading word, protocol supplementary concept word, rare disease supplementary concept word, unique identifier, synonyms] | 343 |
| 7 | exp Sepsis/ | 111801 |
| 8 | exp Meningitis/ | 53216 |
| 9 | exp Meningococcal Infections/ | 10559 |
| 10 | Cerebrospinal Fluid/ | 17871 |
| 11 | "cerebrospinal fluid infection*".mp. [mp=title, abstract, original title, name of substance word, subject heading word, floating sub-heading word, keyword heading word, protocol supplementary concept word, rare disease supplementary concept word, unique identifier, synonyms] | 65 |
| 12 | 1 or 2 or 3 or 4 | 3453 |
| 13 | 5 or 6 or 7 or 8 or 9 or 10 or 11 | 943005 |
| 14 | 12 and 13 | 150 |
| 15 | limit 14 to "all child (0 to 18 years)" | 19 |
